# Time-resolved molecular dynamics of single and double hydrogen migration in ethanol

**DOI:** 10.1038/s41467-019-10571-9

**Published:** 2019-06-27

**Authors:** Nora G. Kling, S. Díaz-Tendero, R. Obaid, M. R. Disla, H. Xiong, M. Sundberg, S. D. Khosravi, M. Davino, P. Drach, A. M. Carroll, T. Osipov, F. Martín, N. Berrah

**Affiliations:** 1 Department of Physics, University of Connecticut, Storrs, CT 06269 USA; 2 Departamento de Química, Módulo 13, Universidad Autónoma de Madrid, 28049 Madrid, Spain; 3 Condensed Matter Physics Center (IFIMAC), Universidad Autónoma de Madrid, 28049 Madrid, Spain; 4 Institute for Advanced Research in Chemical Sciences (IAdChem), Universidad Autónoma de Madrid, 28049 Madrid, Spain; 5 LCLS, SLAC National Accelerator Laboratory, Menlo Park, CA 94025 USA; 6 Instituto Madrileño de Estudios Avanzados en Nanociencia (IMDEA-Nano), Campus de Cantoblanco, 28049 Madrid, Spain; 7 Donostia International Physics Center (DIPC), Paseo Manuel de Lardizabal 4, 20018 Donostia-San Sebastián, Spain

**Keywords:** Atomic and molecular interactions with photons, Chemical physics

## Abstract

Being the lightest, most mobile atom that exists, hydrogen plays an important role in the chemistry of hydrocarbons, proteins and peptides and most biomolecules. Hydrogen can undergo transfer, exchange and migration processes, having considerable impact on the chemical behavior of these molecules. Although much has been learned about reaction dynamics involving one hydrogen atom, less is known about those processes where two or more hydrogen atoms participate. Here we show that single and double hydrogen migrations occurring in ethanol cations and dications take place within a few hundred fs to ps, using a 3D imaging and laser pump-probe technique. For double hydrogen migration, the hydrogens are not correlated, with the second hydrogen migration promoting the breakup of the C–O bond. The probability of double hydrogen migration is quite significant, suggesting that double hydrogen migration plays a more important role than generally assumed. The conclusions are supported by state-of-the-art molecular dynamics calculations.

## Abstract (web summary)

Chemical dynamics in molecules involve particle migration and bond rearrangement. Here the authors show single and double hydrogen migration in ethanol cations and dications that are generated by using intense laser pulse interaction with a jet of ethanol molecules.

## Introduction

Intramolecular hydrogen migration is a natural process that plays a crucial role in many biological, chemical, and physical phenomena^1–3^ and is a topic of current research efforts in fuel cells^4^, proteomics^5,6^, and combustion chemistry^7^. Growing in popularity for its success in determining protein structure is hydrogen exchange combined with mass spectroscopy^8^. In deuterium labeling coupled with mass spectroscopic studies used for determining unknown protein and peptide structures, H/D scrambling or migration induced by the excitation/ionization source can lead to an erroneous interpretation of the measured spectra^5,9^. Thus, there is an increasing need to combine highly differential experimental measurements with realistic theoretical modeling that can anticipate if hydrogen migration is likely to occur under the chosen experimental conditions.

It is known that hydrogen migration can be induced by exciting a molecule in many ways, as illustrated by mass or luminescence spectra resulting from electron impact ionization^10^, X-ray absorption^11^, and ionization following ion collisions^12^. A key tool for understanding hydrogen migration dynamics, developed over the course of the past few decades is ultrafast, strong-field laser induced ionization and dissociation of small hydrocarbons, including the simplest one, acetylene^13–17^, and other polyatomic molecules, such as methanol^18^, ethylene glycol^19^, and acetonitrile^20^. Ultrashort VIS/NIR laser pulses with ~10^14^ W/cm^2^ intensities can singly or doubly ionize molecules in the gas phase, initiating hydrogen migration. In time-resolved studies, a second laser pulse with variable time delay is used to interrogate the molecular motions by further ionization, leading to Coulomb explosion. Hydrogen migration dynamics are thus revealed through detection of the produced ion fragments. Fast nuclear dynamics such as single hydrogen migration, which unfolds on a time scale ranging from a few tens of fs to several hundreds of fs in simple hydrocarbons, have been resolved using such a pump and probe technique^14–16^.

Several previous studies have considered the formation of H_2_^+^ and H_3_^+^ from a variety of precursor molecules, which requires the displacement of more than one hydrogen atom^19,21–23^. In many of these cases, the H_2_^+^ and H_3_^+^ are formed from hydrogen atoms that are close to each other in the molecule (for example, the two or three H atoms bonded to the same atomic center), and therefore are not due to the migration of H atoms from one molecular site to another. Meanwhile, there have been many studies showing that H migration can also play a role in the formation of H_2_^+^ and H_3_^+^ ions^21,24,25^. A clear case of this is the observation of H_3_^+^ after irradiating allene, which has two H atoms at both ends of the molecule and thus at least one H atom must first migrate across the carbon chain^13^.

The studies on the molecular hydrogen ion formation have introduced mechanisms classified as roaming and scrambling. Scrambling occurs when two or more H’s exchange bonding sites in the molecule^25–27^. Roaming describes the case when one H or H_2_ appears to be dissociated from the rest of the molecule, but instead of continuously moving away, it returns. Upon return, it can abstract another H^28^. The scrambling and roaming mechanisms are energetically unfavorable and kinematically unlikely, as confirmed by applications of the Rice–Ramsperger–Kassel–Marcus (RRKM) theory to small hydrocarbons^23,27^.

In the present study, we focus on the observation of H_2_O^+^ and H_3_O^+^ ions, which, in the case of ethanol, are prominently produced and requires the migration of one or two H atoms from the molecular carbon backbone to the OH functional group. Therefore, formation of H_2_O^+^ and H_3_O^+^ differs from that of molecular hydrogen ions in that not all the H atoms can be originally bonded to the same atomic center. Another interesting point is that they make a new bond with the oxygen, versus with each other, undergoing different transition state(s) to reach the final states. H_3_O^+^ ions have already been observed in earlier mass-spectrometry experiments and theory on small alcohols^10,29–32^, although the formation of H_3_O^+^ was found to be insignificant in methanol^21^. Thanks to our combination of 3D imaging and fs laser pump-probe technology, here we go a significant step farther and extract temporal information on the formation of these ions, which gives us direct access to the mechanisms of double hydrogen migration and the corresponding time scales. For completeness and as a reference, we also consider the cases where no and single hydrogen migration reactions occur. Apart from its intrinsic interest to understand the mechanisms of double hydrogen migration, ethanol is also important in many everyday applications including, for instance, as an antiseptic cleaning agent, as an additive in gasoline, and consumption in alcoholic beverages. It is also used as a solvent for nanoparticles^33^ and in fuel cells^4^.

**Fig. 1:**
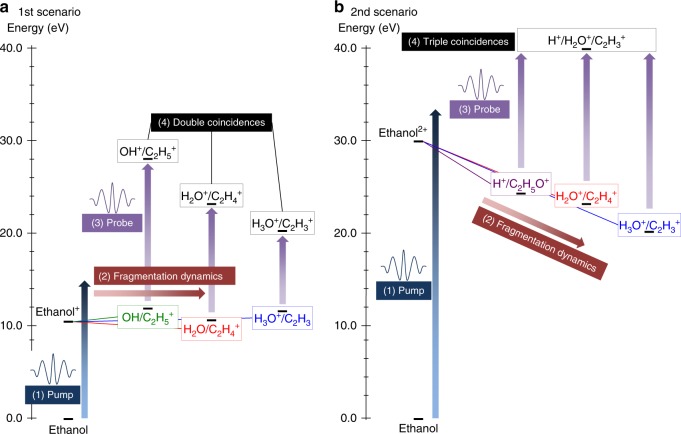
Fragmentation dynamics scenarios. Schematic of the two scenarios investigated with the pump-probe measurements and molecular dynamics calculations, where SHM and DHM leading to H_2_O^+^ and H_3_O^+^ formation, respectively, in ethanol are involved. In the first scenario (**a**) the fragmentation dynamics occur in the cation, whereas for the second scenario (**b**), they occur in the dication. Relative energies for the exit channels are given with respect to the neutral ethanol, computed at the B3LYP/6-31++G(d,p) level of theory

In the following, to track the hydrogen migration reactions that result in H_2_O^+^ and H_3_O^+^ fragments, we follow the ideas set forth by femtochemistry, and use two ultrashort laser pulses in a pump-probe scheme to time-resolve each migration process^34^. There are two scenarios on which we focus our studies, outlined in Fig. 1. In the first scenario (Fig. 1a), we track the dynamics occurring mainly in the ethanol cation, which is experimentally monitored (with the probe pulse) via promotion to the dication, leading to Coulomb explosion and the coincident detection of OH^+^ + C_2_H_5_^+^ (no hydrogen migration, NHM), H_2_O^+^ + C_2_H_4_^+^ (single hydrogen migration, SHM), and H_3_O^+^ + C_2_H_3_^+^ (double hydrogen migration, DHM). In the second scenario (Fig. 1b), our premise is that the pump laser pulse mainly populates the dication, whose fragmentation dynamics are experimentally probed via the triple coincidence channel H^+^ + H_2_O^+^ + C_2_H_3_^+^. This triple coincidence channel is fed by three possible pathways, as the H^+^ can be ejected first, come from the C-containing fragment following the formation of the water ion, or come from the O-containing fragment following formation of the hydronium ion. Our experimental approach allows us to disentangle these three paths.

## Results

### Delay-dependent kinetic energy release

**Fig. 2:**
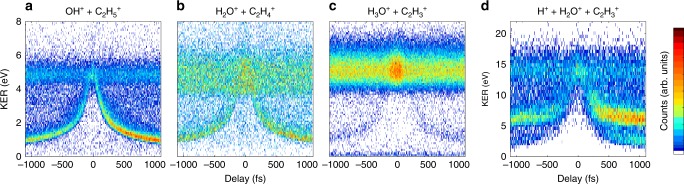
Molecular dynamics for the selected channels. Pump-probe delay-dependence of the KER for the channels **a** OH^+^ + C_2_H_5_^+^, **b** H_2_O^+^ + C_2_H_4_^+^, **c** H_3_O^+^ + C_2_H_3_^+^, and **d** H^+^ + H_2_O^+^ + C_2_H_3_^+^. For positive delay, the pump and probe pulses have an intensity of 4.2 × 10^14^ and 6.7 × 10^14^ W/cm^2^, respectively. For the negative delays, the pump and probe pulses reverse roles. The time-independent features at KER ~5 eV for (**a**–**c**) and ~13 eV for (**d**) are mainly due to interaction with only one of the pulses

Figure 2a–c shows the dependence of the KER on the pump-probe delay for the NHM, SHM, and DHM double coincidence channels. Figure 2d displays the delay-dependence of the KER for the triple coincidence channel, H^+^ + H_2_O^+^ + C_2_H_3_^+^. Each channel displays KER features that clearly change with the delay. Note that for delays close to 0, the pump and probe pulses overlap, giving rise to interferences in the recorded signal, and therefore are largely excluded from further analysis/interpretation.

### Double coincidences

**Fig. 3:**
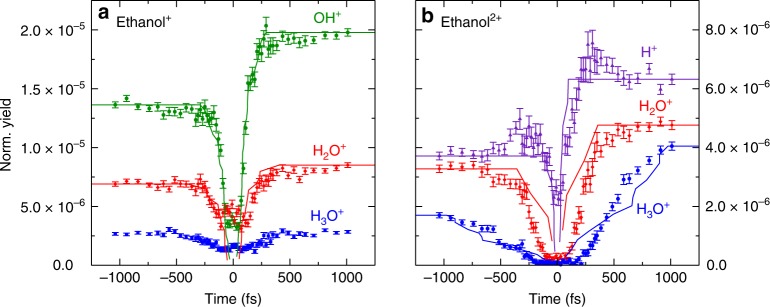
Channel yield versus time. Normalized yield as a function of time for the time-dependent features for the **a** NHM (green, 0–4 eV KER), SHM (red, 0.5–3.6 eV KER), and DHM (blue, 0.5–3.6 eV KER) double coincidence channels and **b** the triple coincidence channel H^+^ + H_2_O^+^ + C_2_H_3_^+^, split into 0–5 eV KER (blue), 5–13 eV KER, inner arch (red), and 5–13 eV KER, outer arch (purple). Experimental error bars are statistical. Theory (solid lines) is carried out for the cation with 10 eV internal energy in (**a**) and the dication with 5 eV internal energy in (**b**)

The features where the KER for NHM, SHM, and DHM shown in panels a, b, and c of Fig. 2 decrease as the pump-probe delay increases are those that follow scenario 1 (see Fig. 1a). What allows us to state that the two-body coincidences mainly reflect the dynamics of the cation is the statistical analysis of the individual channels compared to their precursor(s), which is elaborated on in the Supplementary information (SI). These time-dependent KER features are then selected to monitor their yield as a function of delay between pump and probe laser pulses. The yield is normalized to the number of pulse pairs for a given delay bin. The resulting curves are presented in Fig. 3a.

To get a deeper insight on the H migration mechanisms, we have performed state-of-the-art ab initio molecular dynamics (AIMD) simulations by using the Atom-Centered Density Matrix Propagation^35–37^ method, ADMP, as implemented in the Gaussian09 Package^38^ (see Methods). Figure 3a shows the calculated number of trajectories as a function of the migration time for ethanol cations with an internal excitation energy of 10 eV for both positive and negative delays. The total number of trajectories in each channel has been scaled such that it matches the measured normalized yield at 1 ps, where it practically reaches its saturation value. We have assigned a migration time to a trajectory that results in H_2_O^+^ by identifying the time at which the distance between the migrating H atom and O goes below 1 Å for the first time. Similarly, times for OH^+^ formation have been assigned whenever the distance between the O atom and the C atom from where it departs is larger than 3 Å. We note that no H_3_O^+^ formation (blue symbols in Fig. 1a) is predicted by theory, which is likely due to the limited number of trajectories considered in the cation calculations. From the known excitation energies of ethanol and the AIMD simulations, we estimate that the typical excitation energy of the molecular cation produced by the interaction with the pump pulse should lie between 10 and 15 eV. These numbers agree with those reported in earlier work using similar laser pulses^39^. The H atoms migrate faster with higher initial internal energy, which should make the single and double H migration timescales a bit shorter for negative delays (the more intense pulse comes first) than for positive delays. We do observe this in the experiment, albeit marginally.

By plotting the ratio of the double coincidence channels, as in Supplementary Fig. 7, we investigate the dependence of the channels on each other. It turns out that the ratios are nearly flat as a function of pump-probe time, with some discrepancies discussed in the SI. This is consistent with a competing reaction model^40^—where the production of one channel does not depend on the other in a sequential way.

### Triple coincidences

To probe the dynamics of the dication in the experiment requires forming the dication with the pump pulse, which means that the probe pulse must further ionize the molecule to the trication, resulting in the three ionic fragments H^+^ + H_2_O^+^ + C_2_H_3_^+^—see scenario 2 presented in Fig. 1b. Strictly speaking one cannot completely discard that the H^+^ + H_2_O^+^ + C_2_H_3_^+^ KER also reflects the dynamics induced by the pump pulse in the singly ionized molecule, since the intensity of the probe pulse is large enough to further ionize the monocation to a triply charged final state. A careful inspection of our pump-only data shows that contribution of the monocation dynamics to the NHM and DHM channels should be at most ∼35%, so that scenario 2 reflects most of the physics discussed below. In contrast, for the SHM channel, our pump-only data suggest that the H^+^ + H_2_O^+^ + C_2_H_3_^+^ KER reflects the dynamics of both the monocation and the dication in almost equal proportions. This should be kept in mind in the discussion of the corresponding mechanisms presented below.

The measured KER as a function of pump-probe delay time is presented in Fig. 2d, which shows two delay-dependent KER features. The lower energy feature (0–5 eV) can be attributed to the case where first H_3_O^+^ is formed, and the probe pulse further ionizes and dissociates it into H^+^ + H_2_O^+^. The higher energy feature (5–13 eV) is fed by two precursor channels: one where the H^+^ comes off first (deprotonation), and one where the H_2_O^+^ is formed and the remaining C_2_H_4_^+^ is further ionized and dissociated. We note that, similar to the double coincidence channels, stronger pump pulses (negative delays) ultimately lead to a lower yield of the migration channels. This may be an indication that tunneling ionization prevails over multiphoton ionization or just be the direct effect of having a weaker probe pulse.

**Fig. 4:**
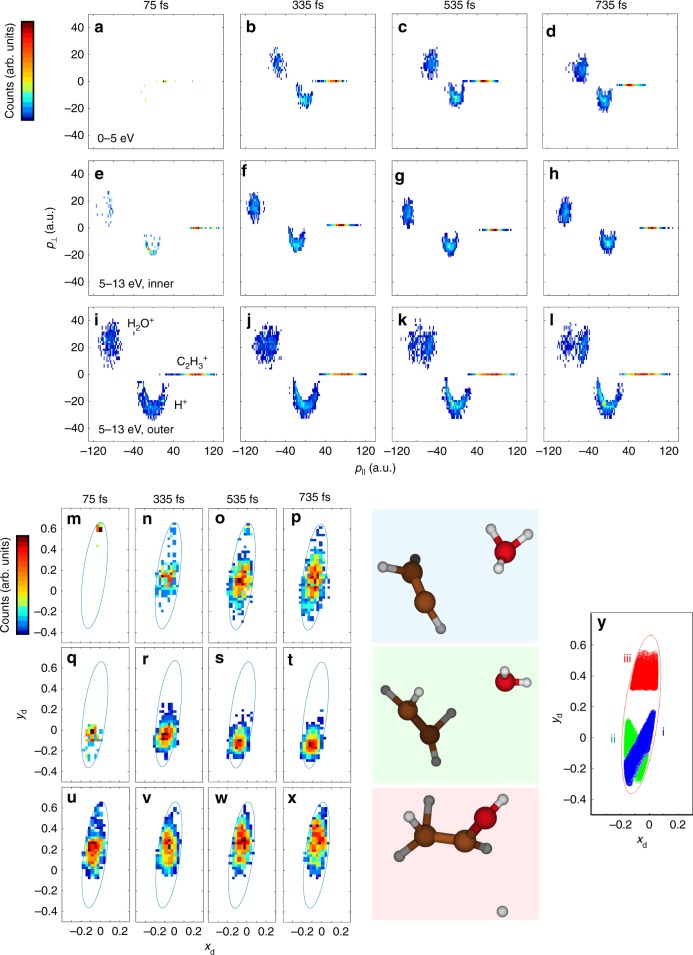
Molecular dynamics for H^+^ + H_2_O^+^ + C_2_H_3_^+^. **a**–**l** Newton and **m**–**x** Dalitz plots for the triple coincidence channel H^+^ + H_2_O^+^ + C_2_H_3_^+^. Panels **a**–**d** and **m**–**p** are for 0–5 eV KER and **e**–**l** and **q**–**x** are for 5 to 13 eV KER. The high KER region is split into **e**–**h** and **q**–**t** which shows the dynamics for the events with *p* < 20 a.u. and **i**–**l** and **u**–**x** for the events with *p* > 20 a.u. The time bins are the same for each column and are from left to right 0–150, 260–410, 460–610, and 660–810 fs (with the center of the time bin denoted). Note that the ellipses are only drawn to help guide the eye, and the color scale, given on the left of the plots range from less counts (bluer) to higher concentration of counts (redder). The blue, green and red shaded boxes show snapshots of the final states reached after the pump step in typical trajectory calculations for the three channels leading to H^+^ + H_2_O^+^ + C_2_H_3_^+^ after the probe step: low KER, high KER inner arch, and high KER outer arch, respectively. Movie frames for the earlier stages of the corresponding fragmentation processes are given in the SI. Panel **y** shows Dalitz plot simulations considering different breakup mechanisms. Features labeled *i* (blue), *ii* (green), and *iii* (red) are for sequential bond breaking with C_2_H_3_^+^, H_2_O^+^, and H^+^ being the first fragment ejected, respectively, and can be related via color to the first step of the breakup processes shown in the shaded boxes of (**b**)

The three-body fragmentation channel lends itself to visualizing the fragment momenta in the molecular frame in the form of a Newton plot^41^, and the energy sharing among the three ions in the form of a Dalitz plot^42^. The Newton plot coordinates are defined such that the C_2_H_3_^+^ ion is always along the positive *x*-axis and the H_2_O^+^ and H^+^ are always in the upper and lower half of the plot, respectively. The results are shown in Fig. 4a–l. The H^+^ momenta $( - p_ \bot )$ for the high KER feature reveals two distinct contributions, forming an inner and outer arch. These two regions are separated into $p = \sqrt {p_ \bot ^2 + p_{||}^2}$< 20 au and >20 au and are plotted individually. While an arch shape in a Newton plot typically represents a sequential fragmentation process, the fact that the H^+^ can come from six different locations in the molecule jumbles everything, which precludes this interpretation.

The Dalitz plot coordinates are defined as

1 $$x_{\mathrm{d}} = \frac{{E_{{\mathrm{C}}_2{\mathrm{H}}_3^ + } - E_{{\mathrm{H}}_2{\mathrm{O}}^ + }}}{{\sqrt 3 \ast {\mathrm{KER}}}}\,{\mathrm{and}}\,{\it{y}}_d = \frac{{E_{{\mathrm{H}}^ + }}}{{{\mathrm{KER}}}} - \frac{1}{3}$$

where *E*_i_ (i = C_2_H_3_^+^, H_2_O^+^, H^+^) are the energies of the individual fragments. To interpret the experimental Dalitz plots shown in Fig. 4m–x, we have simulated the different sequential breakup processes by using a random selection of initial conditions and generated the corresponding Dalitz plot by assuming that all these processes contribute equally, displayed in Fig. 4y.

## Discussion

The Newton and Dalitz plots show a considerable dependence on the time delay for each of the pathways leading to H^+^ + H_2_O^+^ + C_2_H_3_^+^. The low KER contribution, attributed to DHM, is very small at small time delay (Fig. 4a). As time increases (Fig. 4b–d), the yield increases, but the shape of the momentum distribution and the magnitude of the momentum itself does not change significantly. The corresponding Dalitz plots (Fig. 4m–p) are compatible with a sequential mechanism in which C_2_H_3_^+^ is ejected first leaving H_3_O^+^ to further fragment (see process i of Fig. 4y), thus suggesting that the second H migration promotes the breaking of the C–O bond. The energy sharing spreads out with time, indicating that the molecule undergoes significant distortions in its molecular geometry.

For the high KER, inner arch of the Newton plot (Fig. 4e–h), the magnitude of the H^+^ total momentum decreases with time delay, suggesting that the distance between the ions increases and there is less Coulomb repulsion at the time of the bond breakage (see SI for a more quantitative analysis). The corresponding Dalitz distributions (see Fig. 4q–t) appear in the lower part of the allowed region and are consistent with sequential breakups where C_2_H_3_^+^ is ejected first leaving H_3_O^+^ to further fragment (see process i of Fig. 4y) or H_2_O^+^ is ejected first leaving C_2_H_4_^+^ to further fragment (see process ii of Fig. 4y). Therefore, this channel can either be attributed to SHM or to DHM, although given the fact that C_2_H_4_^+^ is less bound than the H_3_O^+^ fragment, most likely we can attribute this section to SHM where the H_2_O^+^ was ejected first. The measured distributions shift to smaller *y*_d_ as the time delay increases, suggesting that H^+^ lies between C_2_H_3_^+^ and H_2_O^+^ when the molecule breaks.

For the high KER, outer arch of the Newton plot (Fig. 4i–l), the general shape of the H^+^ momentum distribution remains the same for all time delays, although the peak in the distribution shifts towards negative *p*_*||*_. Furthermore, the H_2_O^+^ distribution shows a bifurcation, where part of the distribution seems to remain unchanged, and part of it moves towards positive *p*_*||*_. The corresponding Dalitz distributions (Fig. 4u–x) mainly cover the upper part of the allowed region, which is consistent with process iii (see Fig. 4y) in which H^+^ is ejected first corresponding to a deprotonation precursor channel. As the time delay increases, the distribution shifts towards larger *y*_d_ (see SI for a more quantitative analysis) suggesting that the molecular geometry becomes more linear, with either C_2_H_3_^+^ or H_2_O^+^ in the middle. The part of the distribution that moves towards positive *p*_||_ is consistent with having C_2_H_3_^+^ in the middle, and the part that does not change much with pump-probe delay is consistent with having H_2_O^+^ in the middle.

At variance with the ethanol monocation, calculations for the dication do lead to H_3_O^+^ fragments. Thus, the calculated trajectories provide information about the three precursor channels and are split accordingly. The calculated yields as functions of migration time are overlaid with the experimental yield, shown in Fig. 3b. Here, the internal energy used in the calculations is 5 eV for both positive and negative delays. The reason why we have chosen a lower value for the internal energy than in the monocation case is that part of the energy provided by the pump laser must be used to eject a second electron from the ethanol molecule. The good agreement between experiment and theory suggests that the most important dynamics are captured by the calculation.

**Fig. 5:**
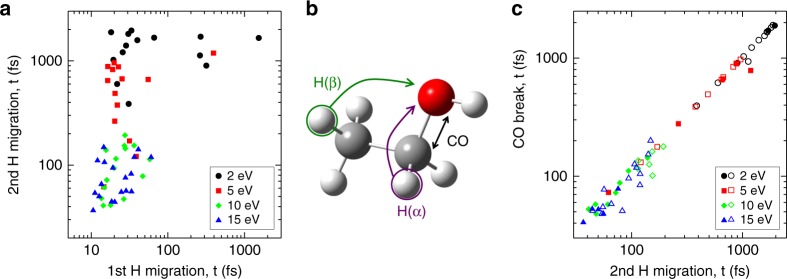
Theory investigation of correlated motions. Correlation of the H migrations themselves and the C–O bond breaking times for trajectories resulting in H_3_O^+^ formation after double ionization. **a** Second versus first H migration time. **b** Scheme of ethanol highlighting the C–O distance (black arrow) and the migration of two H atoms from C_α_ (purple arrow), and C_β_ (green arrow). **c** Correlation between the second H migration and the C–O bond break for H’s originating at the adjacent (C_α_, full symbols) or terminal (C_β_, open symbols) carbon atoms

We can now try to answer the question: how does the double H migration unfold? To begin with, we investigate if there is a correlation between H migration times for the first and second individual migrations for those dication trajectories leading to H_3_O^+^, as shown in Fig. 5a. The large scatter in the plot suggests that the second H migration takes place rather independently of the first migration. This finding is consistent with the experimentally determined time-independent ratios for the double coincidence channels (see Supplementary Fig. 7).

We have also analyzed the AIMD simulation results testing for possible correlations between the time for the first and/or second H migration and the time for the carbon–oxygen bond cleavage (for the trajectories of doubly charged ethanol leading to H_3_O^+^). We observe that there is no correlation between the first H migration and the breaking of the C–O bond. However, as shown in Fig. 5c, there is a clear linear correlation between the second H migration and the breaking of the C–O bond. We thus conclude that the second H migration and the C–O break follows a concerted mechanism, that is, the second migration drives the breakup of the C–O bond. This can be understood in terms of Lewis’s octet rule: after migration of the first proton from the molecular backbone to the O atom, which preserves the electronic octet and locates the positive charge around the latter atom, accommodation of a second H atom (proton + electron) is only possible if the C–O bond breaks, so that one electron leaves its place in the O octet to the incoming electron (the one coming with H).

Another interesting finding is that there is no apparent preference whether the second H comes from C_α_ or C_β_ (see Fig. 5b for the distinction between α and β). As can be seen in Fig. 5, the same behaviors are observed for a wide range of internal energies (from 2 to 15 eV), not only for the internal energy used to evaluate the rate presented in Fig. 3. This points to the generality of the above mechanisms, which may be relevant in other contexts, as there are indications that H migration can drive peptide bond breaking in peptide chains^43^.

In summary, we have combined ultrafast time-resolved imaging techniques and ab-initio molecular dynamics calculations to investigate single and double H migration in ethanol cations and dications. Good agreement between the experimentally measured and calculated yields as a function of pump-probe delay time was achieved, setting a new benchmark standard for studying the ultrafast nuclear dynamics of complex molecules.

The timescales for single and double H migration to form H_2_O^+^ and H_3_O^+^, respectively, is on the order of several hundred fs to ps under the present conditions of the study. Experimentally measured channel yield ratios as a function of pump-probe delay, as well as the AIMD simulations of the ethanol dication, indicate that the two H migrations needed to form H_3_O^+^ are not correlated with each other, and do not show a preference for where they originate from in the molecule. Furthermore, the migration of the second H is concerted with the breaking of the C–O bond.

We hope that our work will help the development of the predictive power necessary to establish routine H migration pathways in complex molecules and contribute new understanding on the timescale of multi-H-migration processes, which are significant for biology, chemistry, and in general for a better understanding of molecular dynamics.

## Methods

### Experiment

The experiment was carried out utilizing two 730 nm (central wavelength), 9 fs laser pulses in a pump-probe configuration with variable time delay between the arrival of the two pulses. The laser pulses were focused on a thin jet of gas phase ethanol inside a cold target recoil ion momentum spectrometer (COLTRIMS), where a static electric field directs ions and electrons to their respective time- and position-sensitive detectors (see Supplementary Fig. 1a). Each separate pair of laser shots provides a new opportunity for an ethanol molecule in the laser focus to undergo light-induced dynamics. Channel selection is accomplished by plotting a 2D histogram of the time of flights of the first and second ions, shown in Supplementary Fig. 1b, which shows (a subset of) the numerous fragmentation pathways, and gating on the pair(s) of ions that conserve momentum for each corresponding channel.

### Theory

Ab initio molecular dynamics (AIMD) simulations were performed by using the Atom-Centered Density Matrix Propagation^35–37^ method, ADMP, as implemented in the Gaussian09 Package^38^. In this method, electronic structure is described quantum mechanically in the framework of the density functional theory—DFT, in particular by using the B3LYP functional^44,45^ in combination with the atomic-centered Gaussian basis set 6-31++G(d,p). In contrast, nuclear motion is assumed to be purely classical and is calculated on the fly by using the forces associated with the instantaneous potential created by the electrons. Following previous theoretical work on coupled electron and nuclear dynamics from excited states in molecules of this size (see, for example ref. ^46–52^), in this work, we assume that the initial excitation energy has already been relaxed to the ground electronic state, which is a reasonable approximation since the investigated migration dynamics are usually slower than the (non-adiabatic) internal conversion processes leading to such energy relaxation. The simulations were performed for the singly and doubly charged ethanol ions resulting from the vertical ionization of the two known conformers of neutral ethanol. For each value of the excitation energy, randomly distributed among the nuclear degrees of freedom, 1000 trajectories were computed. To ensure the adiabaticity of the dynamics, we have imposed a time step of ∆t = 0.1 fs and a fictitious electron mass of 0.1 amu. The maximum propagation time was 3 ps. More details are given in the SI.

## Supplementary information

Supplementary Information

## Data Availability

The data that support the plots within this paper and other findings of this study are available from the corresponding author upon reasonable request.
