# Supplementary material for: Time-resolved molecular dynamics of single and double hydrogen migration in ethanol
Source: Nat Commun. 2019 Jun 27;10:2813. doi: 10.1038/s41467-019-10571-9 (PMC6597707; doi:10.1038/s41467-019-10571-9)
Supplement: Supplementary file 1 — Supplementary Information [file 41467_2019_10571_MOESM1_ESM.pdf]

Supplementary Information:

Supplementary Methods:

The experiment was carried out at the University of Connecticut using a commercially available amplified laser system (Coherent), and a commercially available experimental chamber (Roentdek). To reach the sub-10 fs pulse duration, the 35 fs, 790 nm, 400 μJ laser pulses are loosely focused at the entrance of a 250 μm diameter hollow core fiber, filled with 1.2 bar Ar (see supplementary figure 1). A set of 10 chirped mirrors and a pair of fused silica wedges, after the fiber, compensate for the dispersion acquired in the setup. The pulses are split into a pump and probe and re-combined via a broadband beamsplitter in a Mach Zehnder-type configuration. The probe pulse encounters a linear delay stage, which is programmed to continuously scan a delay range of +/-1.1 ps, with 2 fs intervals, staying 0.3 s at each interval. The total run time was 6 days using the laser at a repetition rate of 10 kHz. The co-linear pulses are introduced to the vacuum chamber through a thin UV-grade fused silica window, where they are back-focused (f=7.5cm) onto the molecular jet (see supplementary figure 1).

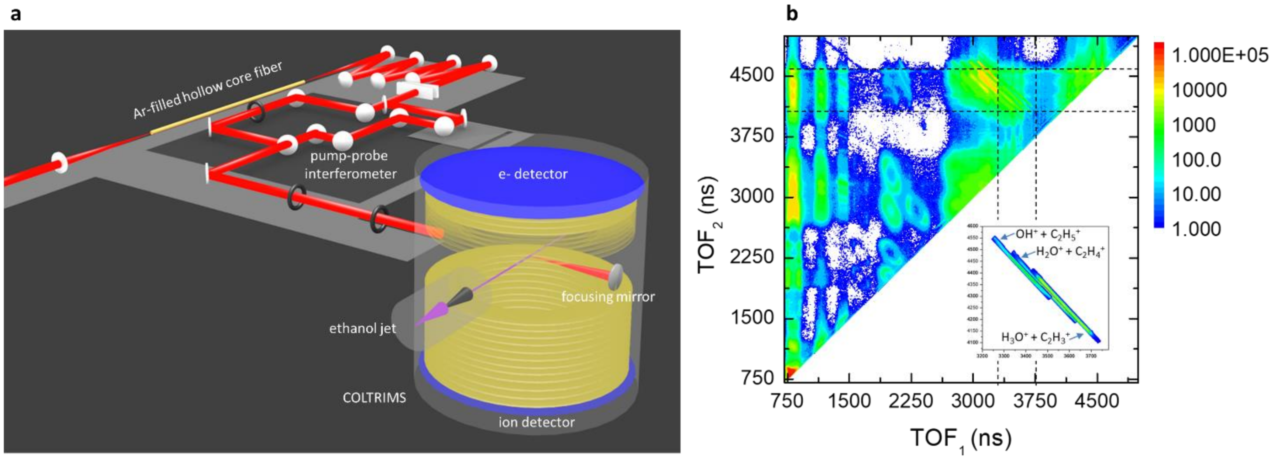

Supplementary Figure 1. Experimental setup and channel identification. (a) Pump-probe and COLTRIMS experimental setup. (b) TOF_1 versus TOF_2 showing the many dissociation channels that result in at least two ions. The inset shows the three two-body breakup channels where the C-O bond was broken. For the inset, the maximum of the linear color scale is 1100 counts.

A liquid ethanol sample is prepared in a sealed container, which is connected to the gas inlet for the COLTRIMS jet. The headspace is evacuated several times to remove the air. The ethanol vapor above the liquid is then expanded into the jet first through a 30 μm diameter nozzle, then a skimmer, providing a ~1 mm diameter, thin jet of ethanol molecules. A static, homogeneous electric field provided by the COLTRIMS spectrometer directs ions to a z-stack microchannel plate detector with a delay line (hex) anode, 27 cm from the interaction region. To collect the highest energy H^+ fragments with full angular acceptance, the spectrometer field was set to about 90 V/cm. This high field and a short extraction region (5 cm) made it impossible to measure electrons with any resolution, although they were also recorded during the experimental run time. The 3D momentum vectors are reconstructed from the time of flight (TOF) and position for each ion [1, 2].

Coincidence channels are identified by plotting the TOF of the first ion that hits the detector versus the TOF of the second ion (subsequent analysis considers all ion pairs that satisfy momentum conservation, not just when the two coincidence ions come as TOF_1 and TOF_2). The three channels presented here, $OH^+ + C_2H_5^+$, $H_2O^+ + C_2H_4^+$, and $H_3O^+ + C_2H_3^+$ are slightly overlapped due to the broad recoil peaks and the difference in mass of the fragments by only a hydrogen. For each channel, constraints are applied which require momentum conservation in all three dimensions to clean the channels from false coincidences.

The Ab Initio Molecular Dynamics simulations were carried out with the Atom Centered Density Matrix Propagation [3-5] method, ADMP, as implemented in the Gaussian09 Package [6]. The ADMP method

is an extended Lagrangian approach in which the classical trajectories are performed by propagating the density matrix. The electronic structure was described in the framework of the density functional theory – DFT, in particular using the B3LYP functional [7, 8] in combination with the atomic centered gaussians basis set 6-31++G(d,p). In order to ensure the adiabaticity of the dynamics we imposed a time step of Δt=0.1 fs and a fictitious electron mass of 0.1 amu.

The simulations were carried out assuming vertical ionization, following the Franck-Condon principle, taking as starting points the two conformers of neutral ethanol (whose geometries were optimized at the same level of theory B3LYP/6-31++G(d,p)). The excitation energy was randomly distributed in each trajectory among the nuclear degrees of freedom, performing the propagation in the electronic ground state of the corresponding ion. For each isomer and each value of excitation energy 500 trajectories were computed. The maximum propagation time was 3 ps; at this point two main analyses were carried out:

-Two atoms have been considered to be bonded if the distance between them is smaller than 3 Å; at larger distances we assume that the atoms belong to separate fragments. The charge in the corresponding fragment is obtained as the sum of the atomic charges in the fragment adopting the Mulliken population scheme [9].

- Migration times for trajectories leading to H_2O^+ or H_3O^+ have been assigned by identifying the time at which the distance between the migrating H atom and O goes below 1 Å for the first time. Times for

OH^+, H_2O^+ or H_3O^+ formation have been assigned by identifying when the distance between the O atom and the C atom is larger than 3 Å.

The computational strategy adopted here was successfully employed to describe the fragmentation dynamics of positively-charged molecules in the gas phase induced in collisions with highly-charged ions – glycine [10], beta-alanine [11], and gamma aminobutyric acid [12] – and in X-ray ionization – thiophene [13]. An example of a full trajectory analysis is shown in supplementary figure 2.

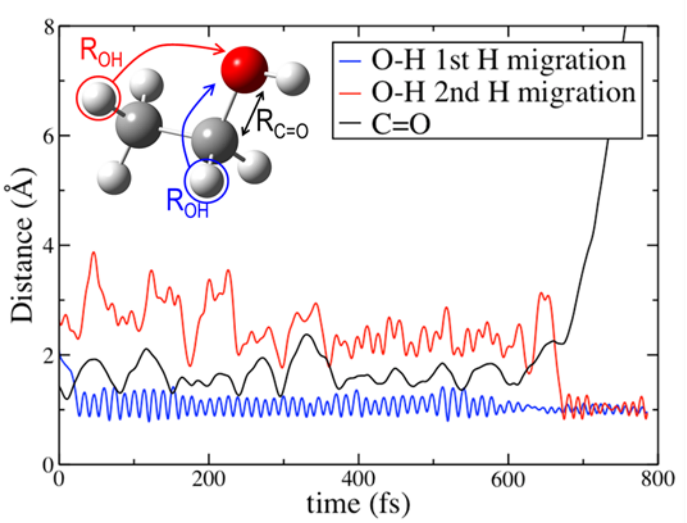

Supplementary Figure 2. Trajectory calculation analysis. Example of the trajectory analysis on an ethanol dication that involves two H migrations for 5 eV initial internal energy. The distances between the oxygen and individual hydrogens are tracked as a function of time. The first time the distance reaches 1 Å (in this example, this happens in the first 10's of fs for the 1st H migration, shown in blue, and after ~650 fs for the 2nd migration, shown in red) is when the H has been considered to be migrated. Also shown in black is the distance between the alpha carbon and the oxygen. The C-O bond breaks immediately following the second H migration.

An illustration of typical trajectories found for the deprotonation, SHM and DHM processes is given in supplementary figure 3.

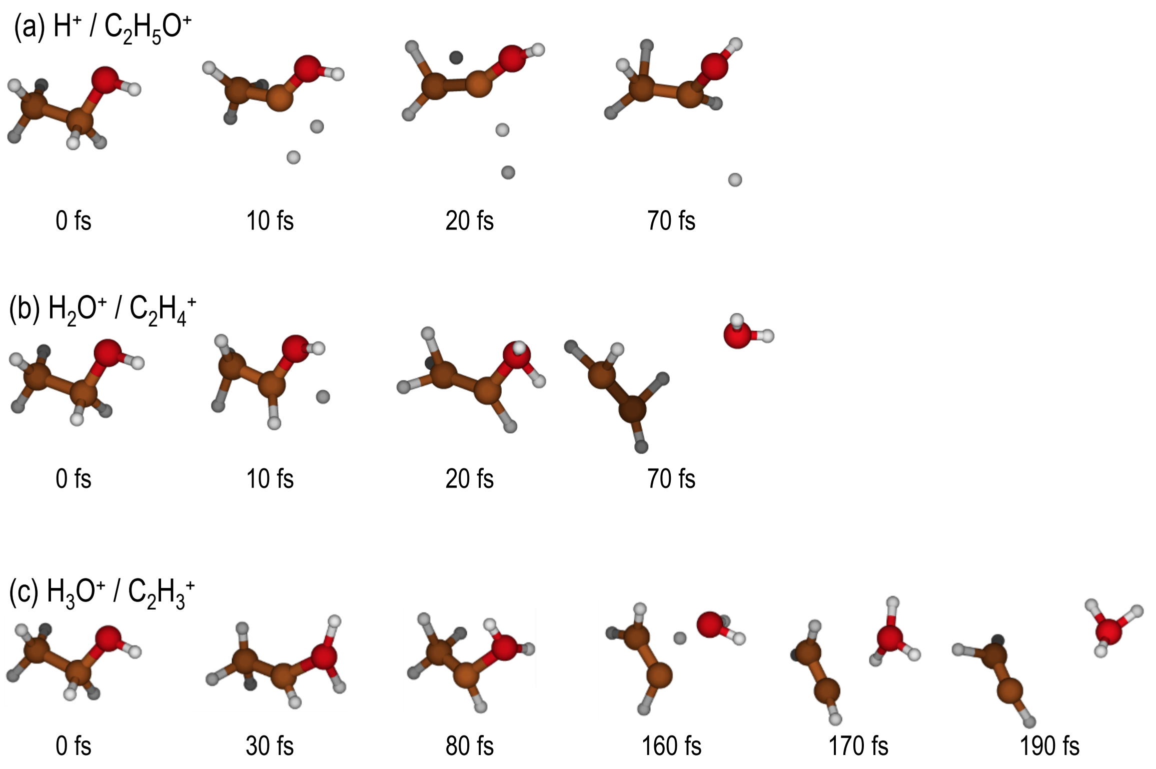

Supplementary Figure 3. Molecular movie snapshots. Movie frames representing the evolution of the ethanol dication along typical trajectories leading to the three precursor channels: a) $H^+ + C_2H_5O^+$, b) $H_2O^+ + C_2H_4^+$ and c) $H_3O^+ + C_2H_3^+$ for the triple coincidence channel $H^+ + H_2O^+ + C_2H_3^+$.

Supplementary Discussion:

Here we discuss the cation and dication contributions to double coincidence and triple coincidence channels. To determine the main ionic state of the molecule following interaction with the pump pulse, we conducted a single pulse study with an intensity ~4×10^{14}W/cm^2 and otherwise the same properties as the laser pulses used in the pump-probe experiment. The yields for the ions and ion pairs following interaction with a single laser pulse are summarized in supplementary table 1 and supplementary figure 4. The ions (full TOF spectrum is plotted in blue in supplementary figure 4) can come with a neutral or charged partner (or partners). To estimate the number of single ions that come with a neutral, we

subtract the number of relevant two-body ion pairs (after considering the detection efficiency of detecting one, ~55%, or two ions, ~30%, estimated by the open area ratio of the detectors). Following Fig. 1 a in the main text, we focus our attention on the $OH^+ + C_2H_5^+$, $H_2O^+ + C_2H_4^+$, and $H_3O^+ + C_2H_3^+$ pairs, respectively. The corresponding yields are also shown in supplementary figure 4. As can be seen, the latter are orders of magnitude smaller than the total individual ion yield. There are no other large contributions of dication pairs that give rise to these single ions. Thus, the number of ion-neutral pairs divided by the number of all single ions provides an estimate of the importance of the cation in the two-body reaction channel, which is above 95% for all three cases.

**Ion (s)**	**Counts**	**Est. % contribution**
$C_2H_5^+$	681360	99.8
$OH^+ + C_2H_5^+$	16660	0.2
$C_2H_4^+$	3359900	99.4
$H_2O^+ + C_2H_4^+$	19550	0.6
H_3O^+	1474280	95.2
$H_3O^+ + C_2H_3^+$	70400	4.8
$H^+ + C_2H_5O^+$	11380	---
$H^+ + H_2O^+ + C_2H_3^+$	14400	14.2

Supplementary Table 1. Individual ion and channel yields. Estimated contributions of cation, dication, and tri-cation to the dynamics involved in the two-body and three-body dissociation channels. The number of counts indicated have been approximately adjusted for the efficiency of detection for the ion or ion pairs, following interaction with a single pulse study having intensity ~4×10^{14}W/cm^2.

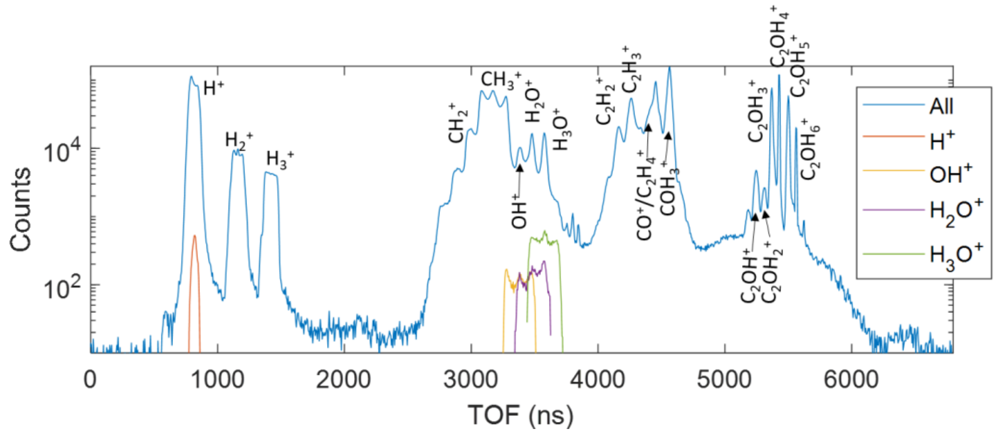

Supplementary Figure 4. Single pulse time-of-flight spectrum. The time-of-flight spectrum from the single pulse study (I ~ 4×10^{14}W/cm^2) is shown in blue. The individual ions indicated in the legend are those that came from double coincidence channels H^+ + $C_2H_5O^+$, OH^+ + $C_2H_5^+$, H_2O^+ + $C_2H_4^+$, and H_3O^+ + $C_2H_3^+$, which represent the number of counts for the channel. The detection efficiency is not taken into account in this plot.

In conclusion, owing to the difference in their yields as a function of pump-probe time delay, the two-body channels mainly reflect the dynamics occurring in the cation and the three-body channel occurring in the dication.

Here we discuss the shift in H^+ momentum for the high KER, inner arch. For the high KER, inner arch, we observe a shift toward lower values in the Newton plot for the H^+ momenta as the pump-probe delay is increased. To further quantify this shift, we plot the H^+ momenta in supplementary figure 5 a for the same delay bins as the Newton and Dalitz plots, as well as a bootstrap analysis of the mean H^+ momenta (see supplementary figure 5 b). Considering all pieces of information, from this H^+ momentum shift, to the Dalitz plot suggesting the H^+ lies between the other two fragments, it seems the second H was well on its way towards the H_2O^+, but upon competing with the rate of dissociation, it does not quite make it before the molecule is dissociated due to Coulomb explosion. The longer duration before the arrival of

the second pulse allows the dication to dissociate to a further extent, such that the Coulomb explosion energy is greatly reduced.

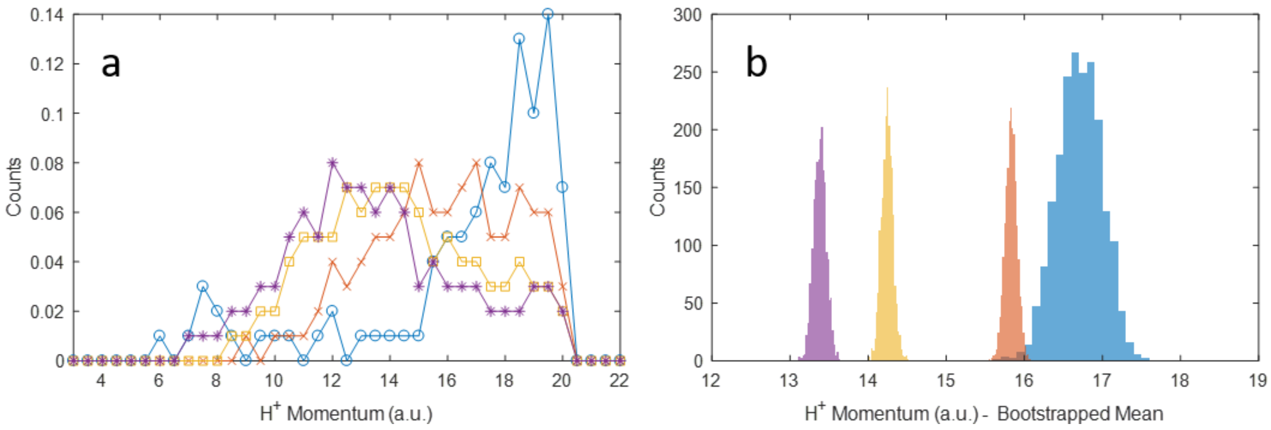

Supplementary Figure 5. Bootstrap analysis for the H^+ momentum. a) H^+ momenta gated on the high KER, inner arch. Blue (75 fs), orange (335 fs), yellow (535 fs), purple (735 fs) symbols clearly show a shift to lower momentum as the pump-probe delay time is increased. b) Bootstrapped mean for the H^+ momenta for the high KER, inner arch. Blue (75 fs), orange (335 fs), yellow (535 fs), purple (735 fs) shaded regions clearly show a shift to lower momentum as the pump-probe delay time is increased. Standard errors for each delay are 0.28, 0.075, 0.074, and 0.082, respectively.

Here we discuss the shift in y_d for the high KER, outer arch. The shift in the center of the y_d distribution from delay=75 fs to 735 fs is about 0.07, for the high KER, outer arch part of the distribution. Given that the full range of y_d goes from -0.36 to 0.66 for the mass distribution of the three fragments, the change in the average value of y_d is ~ 7%. While the shift is perhaps only subtle in a histogram of the counts for y_d (supplementary figure 6 a), bootstrapping the average y_d shows a clear shift, as shown in supplementary figure 6 b.

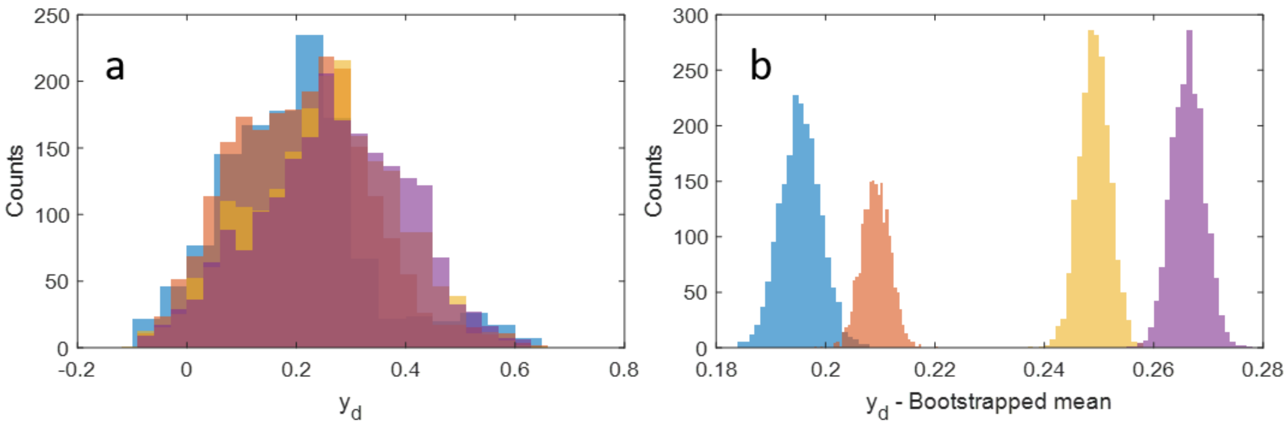

Supplementary Figure 6. Bootstrap analysis for y_d. a) Histogram and b) bootstrapped mean of the vertical axis of the Dalitz plot, y_d, gated on the high KER, outer arch. Blue (75 fs), orange (335 fs), yellow (535 fs), purple (735 fs) distributions show an overall shift to higher y_d as the pump-probe delay time is increased. Standard errors for each delay are 0.0037, 0.0026, 0.0028, and 0.0030, respectively.

Here we discuss the competing reaction mechanism. In order to determine if the channels have any dependence on each other, we have plotted the ratio of the channel yields as a function of pump-probe delay time, shown in supplementary figure 7. The ratios are almost constant across the whole range within the error bars. The constant ratios suggest that these three reaction channels can be modeled as competing reactions without any correlations between the channels. Here the chemical kinetics which describe the yield of each channel as a function of time look like:

$$[\mathrm{C_2H_5OH^{2+}}] = e^{-k_s t} \quad (1)$$

$$[\mathrm{NHM}] = \frac{k_{\mathrm{NHM}}}{k_s}\left[1 - e^{-k_s t}\right] \quad (2)$$

$$[\mathrm{SHM}] = \frac{k_{\mathrm{SHM}}}{k_s}\left[1 - e^{-k_s t}\right] \quad (3)$$

$$[\mathrm{DHM}] = \frac{k_{\mathrm{DHM}}}{k_s}\left[1 - e^{-k_s t}\right] \quad (4)$$

where k_s is the sum of the rate constants (k_i, i = NHM, SHM, DHM). Taking the ratio of any two yields for [NHM], [SHM], and [DHM], one is left with a ratio of the rate constant for those channels. The slight slopes on the red and black curves for ~-250 to ~-50 fs and ~+50 to ~+250 fs (ignoring the points around 0 delay, where the pump and probe pulses overlap) are likely due to the incompleteness of the picture, as there are many other reaction channels that may play a role, so only the large features of the plot should be considered.

Possible reasons for the observed deviations from a flat ratio include: i) contaminations from reactions occurring on the dication, mixing the fragmentation dynamics of the singly charged and the doubly charged ions, and ii) the fact that the other fragmentation channels are not taken into account in the simple kinematical model presented in the supplementary eq. 1-4. Some of the missing channels may follow different dynamics, for example, sequential fragmentation instead of competing fragmentation, and this would introduce deviations from a flat behavior in the ratios between the NHM, SHM and DHM yields (see [14]).

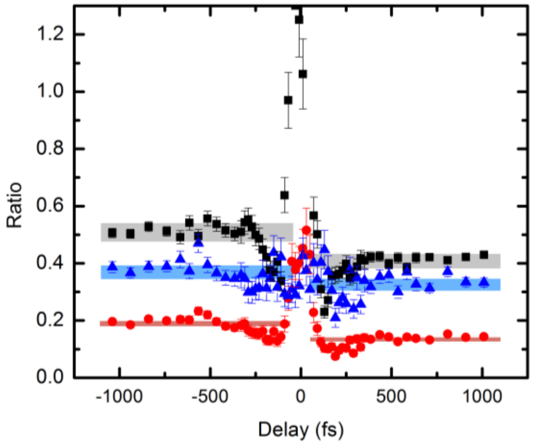

Supplementary Figure 7. Experimental ratios of the channel yields. Ratio of experimental channel yields as a function of pump-probe delay (black squares, blue triangles, and red circles denote the [SHM]/[NHM], [DHM]/[SHM], and [DHM]/[NHM] ratios, respectively). Experimental error bars are

statistical. Shaded regions correspond to the ratios of the rate constants determined from the exponential fitting of the channel yields.

To extract the rate constants from this model, which is the quantity that can be most likened to a H migration time, the three equations (2)-(4) can be used to fit the three experimentally measured normalized yields using a multi-parameter fitting software, where all three k's are determined by the simultaneous fit to the three curves. The resulting formation times ($1/k_\mathrm{i}$) from this procedure are summarized in supplementary table 2, with NHM needing around 200 fs, SHM needing several hundred fs, and DHM needing just upwards of 1 ps. We emphasize that these numbers only have qualitative value, as obtaining accurate values of the rate constants requires taking into account all other competing channels.

**Channel**	**KER (eV)**	**Delay sign**	**Formation time (fs)**
$OH^+ + C_2H_5^+$	0 – 4	+	220 +/- 10
$OH^+ + C_2H_5^+$	0 – 4	-	190 +/- 10
$H_2O^+ + C_2H_4^+$	0.5 – 3.6	+	540 +/- 30
$H_2O^+ + C_2H_4^+$	0.5 – 3.6	-	370 +/- 20
$H_3O^+ + C_2H_3^+$	0.5 – 3.6	+	1645 +/- 95
$H_3O^+ + C_2H_3^+$	0.5 – 3.6	-	1000 +/- 55

Supplementary Table 2. Channel formation times. NHM, SHM, and DHM channels and their experimental formation times determined from the rate constants of a multiparameter, simultaneous fit to the delay dependence of the channel yields for the KER ranges indicated. For positive delays, the probe is more intense than the pump. The role of the pump and probe pulses are reversed for negative

delays. Error bars are estimated from propagating the error of the *k* values given by the fitting procedure with Origin [15].

Here we discuss the effect of the initial internal energy. To check how sensitive the calculated fragmentation yields are to the initial internal energy, in the case of SHM we have performed additional calculations for an internal energy of 15 eV. The results are shown in supplementary figure 8 in the region of negative time delays, where the more intense pulse comes first and therefore the system is expected to have a higher internal energy. For consistency, in the region of positive time delays, where the less intense pulse comes first, the figure shows the results obtained for 10 eV. As can be seen, the results for 15 eV are still in reasonable agreement with experiment.

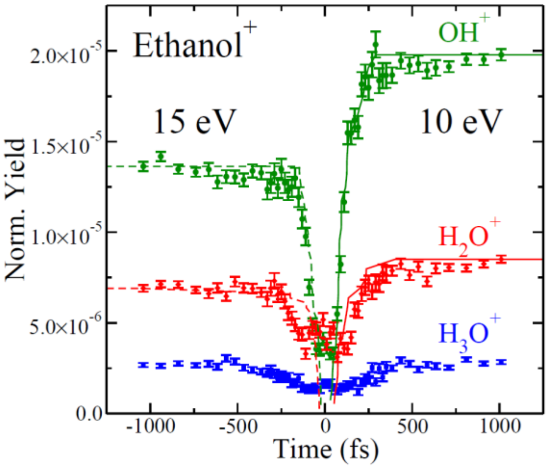

Supplementary Figure 8. Channel yield versus time – importance of internal energy. Normalized experimental yield as a function of time for the time-dependent features for the NHM (green symbols, 0-4 eV KER), SHM (red symbols, 0.5-3.6 eV KER), and DHM (blue symbols, 0.5-3.6 eV KER) double coincidence channels in comparison with the calculated yields for an internal energy of 15 eV (negative delays, dashed lines) and 10 eV (positive delays, solid lines). Experimental error bars are statistical.

Supplementary References:

1. J. Ullrich, R. Moshammer, A. Dorn, R. Doerner, L. Ph. H. Schmidt, H. Schmidt-Boecking, Recoil-ion and electron momentum spectroscopy: reaction microscopes. *Rep. Prog. Phys.* **66**, 1463-1545 (2003).

2. R. Doerner, V. Mergel, O. Jagutzki, L. Spielberger, J. Ullrich, R. Moshammer, H. Schmidt-Boecking, Cold target recoil ion momentum spectroscopy: a ‘momentum microscope’ to view atomic collision dynamics. *Physics Reports* **330**, 95-192 (2000).

3. S. S. Iyengar, H. B. Schlegel, J. M. Millam, G. A. Voth, G. E. Scuseria, M. J. Frisch, Ab initio molecular dynamics: Propagating the density matrix with Gaussian orbitals. II. Generalizations based on mass-weighting, idempotency, energy conservation and choice of initial conditions. *J. Chem. Phys*. 115, 10291-10302 (2001).

4. H. B. Schlegel, J. M. Millam, S. S. Iyengar, G. A. Voth, G. E. Scuseria, A. D. Daniels, M. J. Frisch, Ab initio molecular dynamics: Propagating the density matrix with Gaussian orbitals. *J. Chem. Phys*. 114, 9758-9763 (2001).

5. H. B. Schlegel, S. S. Iyengar, X. Li, J. M. Millam, G. A. Voth, G. E. Scuseria, M. J. Frisch, Ab initio molecular dynamics: Propagating the density matrix with Gaussian orbitals. III. Comparison with Born-Oppenheimer dynamics. *J. Chem. Phys.* 117, 8694-8704 (2002).

6. Gaussian 09, Revision E.01, M. J. Frisch et al., Gaussian, Inc., Wallingford CT, (2013).

7. A. D. Becke, Density-functional thermochemistry. III. The role of exact exchange. *J. Chem. Phys*. **98**, 5648-5652 (1993).

8. C. Lee, W. Yang, R. G. Parr, Development of the Colle-Salvetti correlation-energy formula into a functional of the electron density. *Phys. Rev. B* **37**, 785-789 (1988).

9. R.S. Mulliken, Electronic population analysis on LCAO-MO molecular wave functions. I. *J. Chem. Phys*. **23**, 1833-1840 (1995).

10. S. Maclot, D.G. Piekarski, A. Domaracka, A. Méry, V. Vizcaino, L. Adoui, F. Martín, M. Alcamí, B. A. Huber, P. Rousseau, S. Díaz-Tendero, Dynamics of glycine dications in the gas phase: Ultrafast intramolecular hydrogen migration versus Coulomb repulsion. *J. Phys. Chem. Lett*. **4**, 3903-3909 (2013).

11. D. G. Piekarski, R. Delaunay, S. Maclot, L. Adoui, F. Martín, M. Alcamí, B.A. Huber, P. Rousseau, A. Domaracka, S. Díaz-Tendero, Unusual hydroxyl migration in the fragmentation of β-alanine dication in the gas phase. *Phys. Chem. Chem. Phys*. **17**, 16767-16778 (2015).

12. D. G. Piekarski, R. Delaunay, A. Mika, S. Maclot, L. Adoui, F. Martín, M. Alcamí, B. A. Huber, P. Rousseau, S. Díaz-Tendero, A. Domaracka, Production of doubly-charged highly reactive species from the long-chain amino acid GABA initiated by Ar^{9+} ionization. *Phys. Chem. Chem. Phys*. **19**, 19609-19618 (2017).

13. E. Kukk, D. T. Ha, Y. Wang, D.G. Piekarski, S. Díaz-Tendero, K. Kooser, E. Itälä, H. Levola, M. Alcamí, E. Rachlew, F. Martín, Internal energy dependence in X-ray-induced molecular fragmentation: An experimental and theoretical study of thiophene. *Phys. Rev. A* **91**, 043417 (2015).

14. I. N. Levine, Physical Chemistry (McGraw-Hill, New York, sixth edition, 2002), pp 521-526.

15. Origin (OriginLab, Northampton, MA).
